# Patients with type 1 diabetes and albuminuria have a reduced brain glycolytic capability that is correlated with brain atrophy

**DOI:** 10.3389/fnins.2023.1229509

**Published:** 2023-10-05

**Authors:** Mark B. Vestergaard, Jens Christian Laursen, Niels Søndergaard Heinrich, Peter Rossing, Tine Willum Hansen, Henrik B. W. Larsson

**Affiliations:** ^1^Functional Imaging Unit, Department of Clinical Physiology and Nuclear Medicine, Copenhagen University Hospital – Rigshospitalet, Glostrup, Denmark; ^2^Steno Diabetes Center Copenhagen, Gentofte, Denmark; ^3^Department of Clinical Medicine, Faculty of Health and Medical Science, University of Copenhagen, Copenhagen, Denmark

**Keywords:** diabetes, brain metabolism, brain glycolysis, HIF-1, lactate, cerebrovascular reactivity

## Abstract

**Introduction:**

Patients with type 1 diabetes (T1D) demonstrate brain alterations, including white matter lesions and cerebral atrophy. In this case–control study, we investigated if a reason for this atrophy could be because of diabetes-related complications affecting cerebrovascular or cerebral glycolytic functions. Cerebral physiological dysfunction can lead to energy deficiencies and, consequently, neurodegeneration.

**Methods:**

We examined 33 patients with T1D [18 females, mean age: 50.8 years (range: 26–72)] and 19 matched healthy controls [7 females, mean age: 45.0 years (range: 24–64)]. Eleven (33%) of the patients had albuminuria. Total brain volume, brain parenchymal fraction, gray matter volume and white matter volume were measured by anatomical MRI. Cerebral vascular and glycolytic functions were investigated by measuring global cerebral blood flow (CBF), cerebral metabolic rate of oxygen (CMRO_2_) and cerebral lactate concentration in response to the inhalation of hypoxic air (12-14% fractional oxygen) using phase-contrast MRI and magnetic resonance spectroscopy (MRS) techniques. The inspiration of hypoxic air challenges both cerebrovascular and cerebral glycolytic physiology, and an impaired response will reveal a physiologic dysfunction.

**Results:**

Patients with T1D and albuminuria had lower total brain volume, brain parenchymal fraction, and gray matter volume than healthy controls and patients without albuminuria. The inhalation of hypoxic air increased CBF and lactate in all groups. Patients with albuminuria had a significantly (*p* = 0.032) lower lactate response compared to healthy controls. The CBF response was lower in patients with albuminuria compared to healthy controls, however not significantly (*p* = 0.24) different. CMRO_2_ was unaffected by the hypoxic challenge in all groups (*p* > 0.16). A low lactate response was associated with brain atrophy, characterized by reduced total brain volume (*p* = 0.003) and reduced gray matter volume (*p* = 0.013).

**Discussion:**

We observed a reduced response of the lactate concentration as an indication of impaired glycolytic activity, which correlated with brain atrophy. Inadequacies in upregulating cerebral glycolytic activity, perhaps from reduced glucose transporters in the brain or hypoxia-inducible factor 1 pathway dysfunction, could be a complication in diabetes contributing to the development of neurodegeneration and declining brain health.

## 1. Introduction

Type 1 diabetes (T1D) can affect the brain and damage both gray and white matter, as evidenced by white matter lesions and cerebral atrophy (Ferguson et al., 2003; Moheet et al., 2015). Patients with T1D also have an increased risk for the development of age-related cognitive problems, Alzheimer’s disease and vascular dementia (Smolina et al., 2015; Shalimova et al., 2019; American Diabetes Association, 2021). The exact cause for this increased risk is not fully established, but multiple mechanisms are possibly involved. A likely cause could be diabetes-related vascular complications. Damage to the blood vessel endothelium from hyperglycemia is a characteristic complication of T1D and is part of the pathophysiology causing diabetic neuropathy, nephropathy, and retinopathy (Rossing et al., 1995; DiMeglio et al., 2018). Endothelium damage reduces the vasodilatory function of the arteries and hinders sufficient delivery of nutrients to the tissue supplied by the vessel. These effects are also observed in brain vessels. Studies have demonstrated reduced cerebrovascular reactivity (CVR) to vasodilative stimuli in patients with T1D (Fulesdi et al., 1997), similar to what is observed in patients with neurodegenerative diseases (Glodzik et al., 2013). Reduced CVR is also associated with gray matter atrophy, cerebral white matter alterations, cognitive decline, and general poorer brain health (Silvestrini et al., 2006; Catchlove et al., 2018; Jensen et al., 2018).

Cerebral glucose metabolism dysfunction could also be a cause for accelerated brain atrophy, and a reduced cerebral metabolic rate of glucose (CMRglu) is a hallmark of Alzheimer’s disease (Jack et al., 2018). Similar to reduced CVR, a failure to upregulate glucose metabolism, when necessary, could lead to energy starvation. The brain primarily depends on glucose to complete the full oxidative pathway for energy production; however, a portion of glucose only goes through oxygen-free glycolysis. An upregulation of glycolysis without a comparable increase in subsequent oxidative phosphorylation will result in excess production of pyruvate, which will be converted to lactate by the lactate dehydrogenase in an equilibrium favoring lactate. This glycolysis pathway is particularly important during neuronal activation, where glycolytic activity is rapidly upregulated, likely to ensure sufficient energy for neurons in the initial phase of activation (Fox et al., 1988). Furthermore, glycolysis is important in the response to hypoxia in the brain, as demonstrated by arterial desaturation causing a quick and robust increase in brain lactate (Vestergaard et al., 2022).

Several mechanisms have been suggested as potential causes for reduced glucose metabolism in relation to the development of neurodegenerative diseases, some of which can be associated with diabetes pathology and insulin depletion. Although the brain does not require insulin to transport glucose into the tissue, insulin receptors still exist in the brain, where insulin plays an important signaling role (Koepsell and De, 2020). Insulin is involved in multiple pathways in the brain, including functions related to synaptic formation, cell proliferation and cognitive function (Havrankova et al., 1978; Plum et al., 2005; Scherer et al., 2021) and regulation of cerebral energy and glucose metabolism (Schell et al., 2021). Insulin is also involved in the response to hypoxia in the brain by increasing hypoxia-inducible factor 1 (HIF-1) (Treins et al., 2002; Doronzo et al., 2006) and by regulating glycolytic activity through the expression and translocation of glucose transporter 3 (GLUT3) (Frazier et al., 2020). Preclinical studies have found that hyperglycaemia reduces GLUT1 and GLUT3 expression in the brain (Wei-kai et al., 2007). Postmortem human studies have also found reduced GLUT in the brains of patients with Alzheimer’s disease (Harik, 1992; Kyrtata et al., 2021), and that carriers of the apolipoprotein E4 gene, who have a significantly higher risk of developing Alzheimer’s disease, have fewer GLUT3 transporters in the brain than noncarriers (Zhao et al., 2017). Overall, these results suggest that patients with diabetes could have inadequacies in upregulating cerebral glycolysis and glucose metabolism due to an absence of insulin signaling or reduced GLUT levels, and such a dysfunction may be related to neurodegeneration and the development of dementia.

Therefore, in the present study, we investigated whether patients with T1D and indications of microvascular damage, evident by albuminuria, have accelerated brain atrophy and whether this can be related to cerebrovascular or glycolytic dysfunction. We examined these functions by using a hypoxic challenge, where the participants inhaled air with reduced oxygen content, resulting in arterial oxygen desaturation. The advantage of using a hypoxic challenge is that we can challenge both the responsiveness of the cerebrovascular system and cerebral glycolytic activity simultaneously. In healthy subjects, the inspiration of moderately hypoxic air stimulates a large increase in CBF to counteract desaturation, and the cerebral metabolic rate of oxygen (CMRO_2_) is maintained (Kety and Schmidt, 1948; Cohen et al., 1967; Vestergaard and Larsson, 2019). If the brain endothelial function of T1D patients is affected, their CVR will likely be reduced compared to that of a control group, which could result in an incapability to maintain CMRO_2_. Additionally, the inhalation of hypoxic air causes a robust increase in cerebral lactate concentration, demonstrating an upregulation of cerebral glycolysis likely as an early adaptive response to protect against hypoxia (Vestergaard et al., 2022). This upregulation of glycolysis occurs even when oxygen metabolism is maintained and is likely triggered by sensing of reduced oxygen pressure in the arterial blood (Vestergaard et al., 2022). A small lactate response will suggest a reduced glycolytic capability. By using hypoxia to challenge cerebral physiology, compared to, for example, a hypercapnic or acetazolamide challenge, we can therefore simultaneously assess whether CVR is impaired, whether a possibly reduced CVR is severe enough to affect oxygen metabolism and whether the glycolytic capability is affected.

We used magnetic resonance imaging (MRI) scanning to examine the participants at normoxia and during the inhalation of hypoxic air while they were lying in the scanner. Using functional MRI sequences and magnetic resonance spectroscopy (MRS), we assessed the effects of hypoxia on CBF, CMRO_2_ and the cerebral lactate concentration.

## 2. Methods

### 2.1. Participants

Patients were recruited from the out-patient clinic at Steno Diabetes Center Copenhagen. The inclusion criterium for TD1 patients was disease duration more than 10 years. Healthy controls were recruited by advertisement in local newspaper and websites. Exclusion criteria for all participants were a history of heart disease, lung disease, neurological disease, symptoms of cerebral artery stenosis, surgery within the last 6 weeks or foreign objects of metal in the body prohibited MRI-scanning.

In total, the study included 33 patients with T1D (17 females, 15 males, mean age: 50.8 years (range: 26–72)) and 19 matched healthy controls (7 females, 12 males, mean age: 45.0 years (range: 24–64)). The patients were divided into groups without (*n* = 22) and with (*n* = 11) albuminuria, defined as a urine albumin creatinine ratio (UACR) above 30 mg/g in two out of three consecutive measurements retrieved from the patient record. Current UACR were additionally measured from most recent urine sample taken in the outpatient clinic before the study visit. Albuminuria is a good indicator of vascular damages and higher allostatic load from diabetes disease and is therefore a useful parameter for dividing the patients into groups of disease severity (Deckert et al., 1989). A summary of the clinical parameters is provided in Table 1.

**Table 1:** Clinical description of the participants.

	Healthy controls	T1D without albuminuria	T1D with albuminuria
Sex [female/male]	7/12	13/9	4/7
Age [years]	45.0 ± 14.0	47.3 ± 12.9	56.0 ± 10.8
Disease duration [years]		28.3 ± 10.5	34.9 ± 17.4
Blood glucose [mmol/l]		9.4 ± 3.8	8.0 ± 1.2
Systolic blood pressure [mmHg]	128 ± 13	133 ± 17	138 ± 15
Diastolic blood pressure [mmHg]	71 ± 10	75 ± 11	80 ± 11
Body mass index [kg/m^2^]	26.1 ± 5.4	25.6 ± 4.2	26.4 ± 3.8
Hemoglobin [mmol/l]	8.9 ± 0.6	8.9 ± 0.7	8.6 ± 0.7
Hemoglobin A1c [mmol/mol]	35.3 ± 3.3	58.6 ± 8.3	54.6 ± 8.8
Current urine albumin creatinine ratio [mg/g]	5.0 [3.3–6.0]	4.0 [3.0–9.0]	28.0 [11.3–65.3]
Estimated glomerular filtration rate [ml/min/1.73 m^2^]	99.0 ± 13.3	104.0 ± 10.9	85.6 ± 24.0
Low density lipoprotein cholesterol [mmol/l]	2.9 ± 1.0	2.3 ± 0.8	1.6 ± 0.5
Serum creatinine [mmol/l]	77.1 ± 13.6	67.2 ± 10.8	83.0 ± 24.8

Continuous parameters are noted as mean ± standard deviation, exempt for urine albumin creatinine ratio which is noted as median [1. quartile – 3. quartile] due to the skewed distribution.

Written informed consent was obtained from the participations prior to examination. The study was approved by the Capital Region of Denmark Committee on Health Research Ethics (H-18036532) and was carried out according to the Declaration of Helsinki.

### 2.2. Experimental design

**Figure 1:**
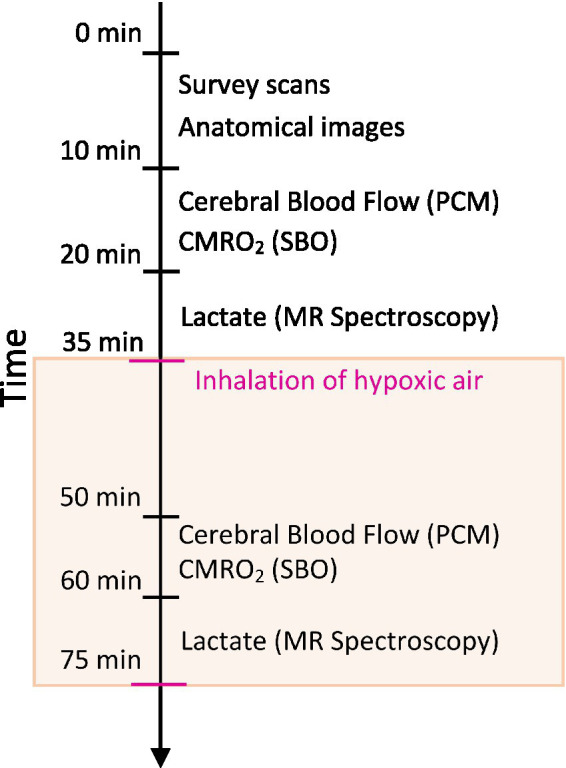
Schematic of the MRI session. High-resolution anatomical scans were acquired to assess brain volumes. Cerebral blood flow (CBF) was measured using a phase contrast mapping (PCM) sequence. The cerebral metabolic rate of oxygen (CMRO_2_) was calculated by Fick’s principle with the measurement of venous oxygen saturation using susceptibility-based oximetry (SBO). Cerebral lactate concentration was measured using MR spectroscopy. Measurements of CBF, CMRO_2_ and lactate were acquired during normoxia with repeated measurements during the inhalation of hypoxic air (12–14% fractional oxygen).

**Figure 2:**
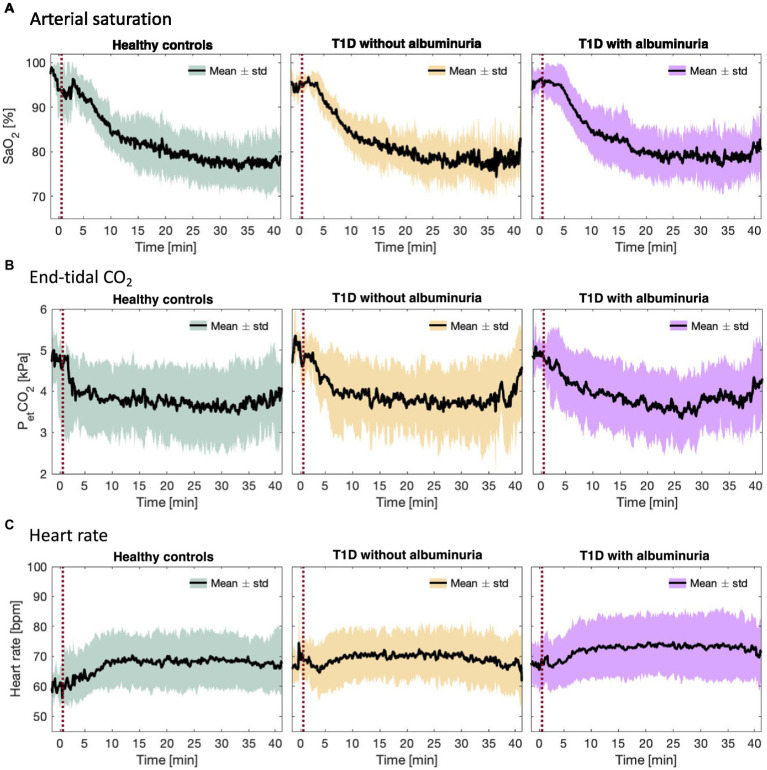
**(A)** Arterial oxygen saturation (SaO_2_), **(B)** end-tidal CO_2_ partial pressure (P_et_CO_2_), and **(C)** heart rate during the inhalation of hypoxic air in the healthy control group and type 1 diabetes (T1D) patients with and without albuminuria. The red line indicates the start of the inhalation of hypoxic air.

Participants first attended an information visit, followed by a visit for blood sampling and an interview about their health status at Steno Diabetes Center Copenhagen. Following this, the participants underwent a single MRI-scan session on another day at the Department of Clinical Physiology and Nuclear Medicine at Rigshospitalet Glostrup. All subjects were MRI-scanned using a Philips 3 T dSTREAM Achieva MRI-scanner (Philips Medical Systems) with a 32-channel phased array head coil. The experiment consisted of a single MRI scan session (Figure 1). Anatomical images were acquired initially in the session. CBF, CMRO_2_ and lactate concentration were measured during normoxia, and the acquisitions were hereafter repeated during the inhalation of hypoxic air with 12–14% fractional oxygen content (Figure 1). During the normoxia measurements, the subjects inhaled atmospheric air from the surrounding environment. For the hypoxia measurements the subjects were fitted with a face mask covering both the mouth and nose while lying in the MRI-scanner. The mask was connected by a wide tube and a one-way valve to an AltiTrainer system (SMTEC, Nyon, Switzerland) that provided the hypoxic air. The heart rate, arterial oxygen saturation (SaO_2_), inspired and expired oxygen partial pressure and expired carbon dioxide partial pressure (PetCO_2_) were measured continuously throughout the scan using a Veris Monitor system (Medrad, Pittsburgh, PA, USA). Blood glucose was self-reported by the patients immediately prior to the MRI scan. The time series of SaO_2_, PetCO_2_ and heart rate during the inhalation of hypoxic air are shown in Figure 2. Blood pressure was measured once while the subjects were lying in the MRI-scanner also using the Veris Monitor system.

### 2.3. MRI sequences

#### 2.3.1. Structural images

**Figure 3:**
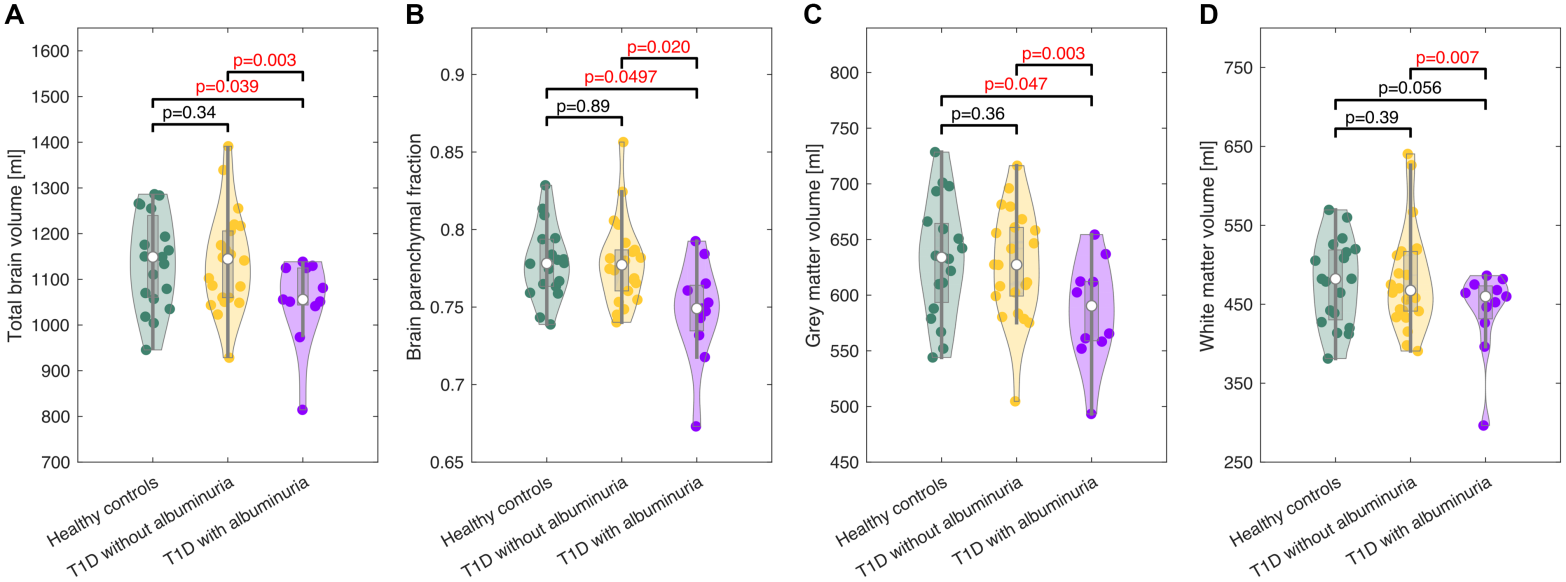
Violin plots of the total brain volume **(A)**, brain parenchymal fraction **(B)**, gray matter volume (C), and white matter volume **(D)** in healthy controls and patients with type 1 diabetes (T1D) with and without albuminuria. The box plots indicate the median, quartiles and 1.5 interquartile range. The truncation of the violins indicates the range of data. Patients with albuminuria had lower total brain and gray matter volumes than healthy controls and patients without albuminuria.

High-resolution structural brain images were acquired by a 3D T1-weighted turbo field echo sequence (echo time (TE) = 5.11 ms; repetition time (TR) = 11.2 ms; flip angle = 8°; voxel size = 0.70 × 0.70 × 0.70 mm^3^). The total brain volume, gray matter volume, white matter volume and brain parenchymal fraction, calculated as the total brain volume to intracranial volume ratio (Figure 3), were estimated through the segmentation of the brain using the FreeSurfer software package (v7.3.2, Martinos Center for Biomedical Imaging, MA, USA) (Fischl, 2012).

#### 2.3.2. Cerebral blood flow

Cerebral blood flow was calculated by measuring the blood flow through the feeding cerebral arteries (the carotids and basilar artery) using velocity-sensitive phase contrast mapping (PCM) MRI (Bakker et al., 1996; Vestergaard et al., 2017). Blood velocity-weighted images were acquired by a velocity-encoding turbo field gradient-echo sequence (1 slice; voxel size = 0.75 × 0.75 × 8 mm^3^; TE = 7.33 ms; TR = 27.63 ms; flip angle = 10°; velocity encoding = 100 cm/s; without cardiac gating). The blood flows in the cerebral arteries were calculated by multiplying the mean blood velocity by the cross-sectional area of regions of interest (ROIs) defining each vessel. The ROIs were manually delineated. We normalized the total blood flow to the brain to the Individual brain volumes to achieve CBF values in ml/100 g/min. Postprocessing was performed using in-house developed software (see code availability statement).

#### 2.3.3. Cerebral metabolic rate of oxygen

CMRO_2_ was calculated by the Fick principle (Equation 1).


(Eq. 1) $${\mathrm{CMRO}}_{2}=\mathrm{Hgb}\cdot \mathrm{CBF}\cdot \left({\mathrm{SaO}}_{2}-{\mathrm{SvO}}_{2}\right)$$

The hemoglobin concentration (Hgb) was measured from venous blood samples. The venous oxygen saturation (SvO_2_) of the blood leaving the brain in the sagittal sinus was obtained using the susceptibility-based oximetry (SBO) MRI technique (Jain et al., 2010, 2011). Susceptibility-weighted maps were calculated from the phase evolution between two echoes acquired using a dual-echo gradient-echo sequence (1 slice, voxel size = 0.688 × 0.688 × 8 mm^3^; TE1 = 8.07 ms; TE2 = 17.77 ms; flip angle = 30°; 5 repeated measures, total duration = 1 min 30 s; SENSE-factor = 2). SvO_2_ was calculated from the phase change in the sagittal sinus and the phase change of immediately adjacent brain tissue from manually delineated ROIs. Aliased phase values in the ROIs were manually corrected. An in-depth discussion of the sequences and postprocessing has been reported previously (Jain et al., 2010; Vestergaard and Larsson, 2019). The postprocessing was performed using in-house developed software (see code availability statement).

#### 2.3.4. Cerebral lactate

Cerebral lactate concentrations were measured by the MRS technique using a water-suppressed point-resolved spectroscopy (PRESS) pulse sequence (TR = 3,000 ms; TE = 288 ms; voxel size = 30 × 35 × 30 mm^3^). The MRS voxel was placed in the precuneus. The sequence was optimized to measure lactate by using a long echo time of 288 ms. Postprocessing and quantification were performed using LCModel (LCModel [Version 6.3-1F], Toronto, Canada). The water peak measured in the spectrum was used to quantify the lactate concentration. The water concentration in the voxel was calculated based on the proportions of gray matter, white matter, and cerebrospinal fluid in the voxel found by tissue segmentations of the anatomical images. Lactate concentrations were corrected for T2 decay using literature values (Wansapura et al., 1999; Träber et al., 2004).

### 2.4. Statistical analyses

Sample size was determined assuming that the lactate response to the hypoxic challenge was the limiting parameter. Power calculation was performed using data from a study with a similar experimental setup to the present study, which found a standard deviation of 10.8% for the lactate response to inspiration of hypoxic air (Vestergaard et al., 2022). The sample size required for a power of 80 and 5% significance level was thus 10 participants per group.

Data postprocessing was performed blinded with regard to subject and measurement (baseline measurement or gas challenge measurement). Values are presented as the mean ± standard deviation for normal distributed parameters and median [1. quartile – 3. quartile] for non-normal distributed parameters. Two-sided *p*-values less than 0.05 were considered significant.

Differences in brain volumes and resting physiology values between healthy controls, T1D patients without albuminuria and T1D patients with albuminuria were tested using linear regression models with group indication as a categorical regressor (Table 2). Sex and age were added as covariates to correct for differences between the groups.

**Table 2:** Summary of the acquired brain volumes and resting brain physiology metrics.

				Group differences		
	Healthy controls	T1D without albuminuria	T1D with albuminuria	Healthy controls vs. T1D without albuminuria	Healthy controls vs. T1D with albuminuria	T1D without albuminuria vs. T1D with albuminuria
**Brain volumes**						
Total brain volume [ml]	1,139 ± 103	1,139 ± 107	1,053 ± 94	CI = [−31; 87], *p* = 0.34	CI = [−139; −4], *p* = 0.039*	CI = [−181; −411], *p* = 0.003*
Brain parenchymal fraction	0.78 ± 0.02	0.78 ± 0.03	0.75 ± 0.03	CI = [−0.02; 0.02], *p* = 0.89	CI = [−0.05; −0.00], *p* = 0.0497*	CI = [−0.05; −0.01], *p* = 0.020*
Gray matter volume [ml]	632 ± 52	628 ± 49	585 ± 45	CI = [−14; 38], *p* = 0.36	CI = [−62; −0.4], *p* = 0.047*	CI = [−75; −17], *p* = 0.003*
White matter volume [ml]	478 ± 54	482 ± 65	441 ± 55	CI = [−10; 49], *p* = 0.39	CI = [−77.6; 1.1], *p* = 0.056	CI = [−106; −19], *p* = 0.007*
**Resting brain physiology**						
CBF [ml/100 g/min]	42.0 ± 4.9	41.3 ± 8.6	39.3 ± 8.6	CI = [−5.0; 3.3], *p* = 0.68	CI = [−6.1; 3.0], *p* = 0.49	CI = [−3.6; 7.5], *p* = 0.47
CMRO_2_ [μmol/100 g/min]	124.8 ± 20.5	130.9 ± 32.8	117.6 ± 23.4	CI = [−13.4; 21.4], *p* = 0.65	CI = [−22.3; 14.8], *p* = 0.68	CI = [−25.8; 21.5], *p* = 0.85
Lactate [mmol/l]	0.53 ± 0.12	0.48 ± 0.10	0.42 ± 0.09	CI = [−0.08; 0.03], *p* = 0.32	CI = [−0.12; 0.02], *p* = 0.14	CI = [−0.11; 0.03], *p* = 0.30

Mean ± standard deviation is reported. *Denotes significant differences. CBF, cerebral blood flow; CI, 95% confidence interval; CMRO_2_, cerebral metabolic rate of oxygen; T1D, type 1 diabetes.

**Figure 4:**
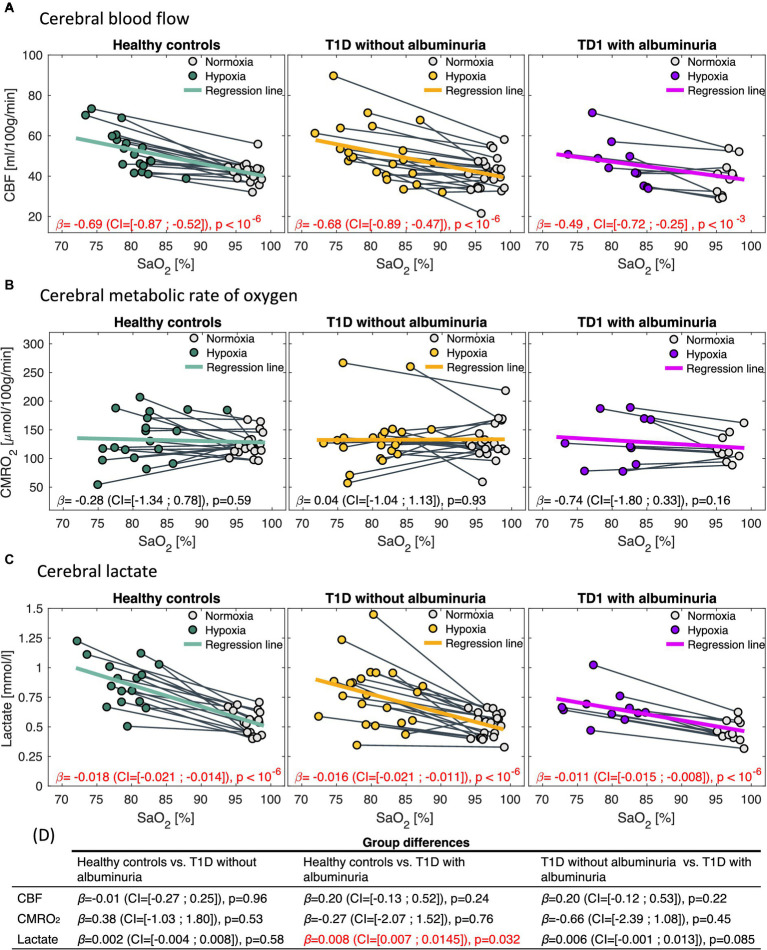
Correlations between arterial oxygen saturation (SaO_2_) and **(A)** cerebral blood flow (CBF), **(B)** cerebral metabolic rate of oxygen (CMRO_2_) and **(C)** cerebral lactate in response to the inhalation of hypoxic air. The first column demonstrates healthy controls, the second column type 1 diabetes (T1D) patients without albuminuria and the third column T1D patients with albuminuria. The regression coefficients [in (ml/100g/min)% , (μmol/100g/min)% and (mmol/l)% for the CBF, CMRO_2_ and lactate analysis, respectively] with 95% confidence intervals (CI) and *p*-values from the regression models is noted in each panel. CBF and lactate increased following the inhalation of hypoxic air in all groups (*p* < 10^−3^). CMRO_2_ was unaffected in all groups. Comparisons between the response to hypoxic challenge between the groups are noted in panel **(D)**. Patients with albuminuria had a significantly (*p* = 0.032) lower lactate response to the hypoxic challenge than the control group.

The effects of the inhalation of hypoxic air on the CBF, CMRO_2_, and lactate concentration were tested by linear mixed regression models with the physiologic measurement as the response variable, SaO_2_ as a fixed effect, and each subject as a random effect (Figure 4). The regression coefficient ( β ) with the 95% confidence interval (CI) and *p*-values from the regressions are noted in each panel in Figure 4. To test whether the responses to hypoxia were different between the groups, we added an interaction term between SaO_2_ and group indication (healthy controls, T1D patients without albuminuria or T1D patients with albuminuria) to the model. The model is demonstrated in equation 2. The significance of the interaction term ( $\beta_{3}$ ) will indicate whether the three groups had different effects on the acquired parameters with regard to oxygen desaturation (Gujarati, 1970). The coefficient and significance of the interaction terms are shown in Figure 4D. Sex and age were added as covariates. The proportion of gray matter in the MRS voxel were additional included as a covariate in the model on lactate response to correct for the different composition of brain tissue in the MRS voxels between subjects.


(Eq. 2) $$\begin{array}{l} Y=\beta_{1}\cdot \mathrm{group}+\beta_{2}\cdot {\mathrm{SaO}}_{2}+\beta_{3}\cdot \mathrm{group}\cdot {\mathrm{SaO}}_{2}+\\ \beta_{4}\cdot \mathrm{Sex}+\beta_{5}\cdot \mathrm{Age}+u \end{array}$$

To account for repeated measurements and differences in baseline values among participants, we included the random effect regressor *u*, which identifies each participant. By including this random effect in the model, we only test the response to the hypoxic challenge and not differences in baseline values (Gujarati, 1970).

**Figure 5:**
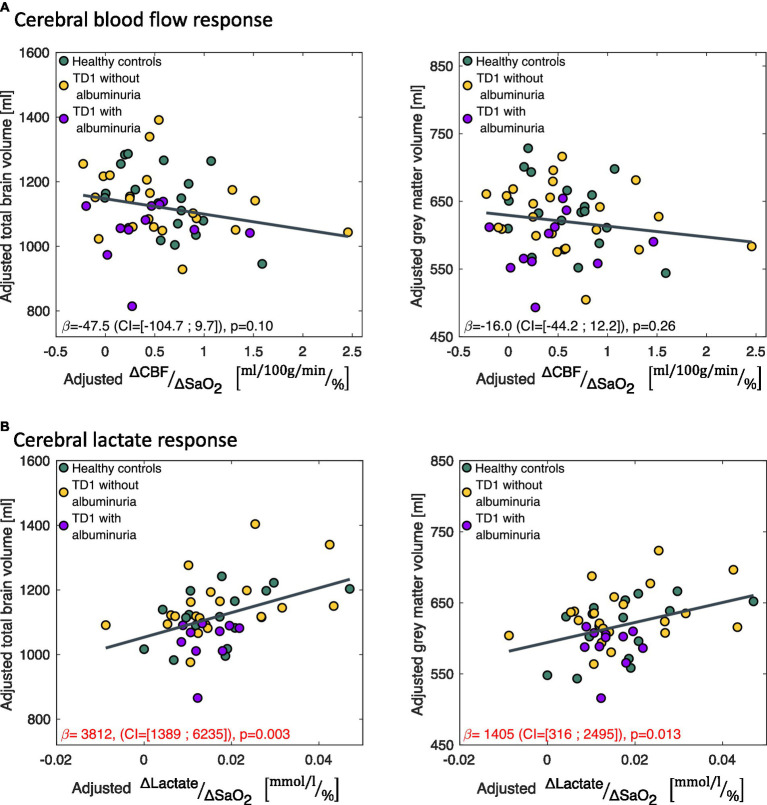
Correlations between the change in **(A)** cerebral blood flow ( ΔCBFΔSaO2 ) and **(B)** cerebral lactate concentration ( ΔLactateΔSaO2 ) in response to the hypoxic challenge and total brain volume and total gray matter volume. ΔLactateΔSaO2 correlated significantly with total brain volume and gray matter volume. The data is presented as partial regression plots with values adjusted for covariates (sex, age, proportion of gray matter in the magnetic resonance spectroscopy voxel).

The correlations between the CBF ( ΔCBFΔSaO2 ) and lactate response ( ΔLactateΔSaO2 ) to the hypoxic challenge and brain volumes were calculated using linear regression models with total brain volume or gray matter volume as the response variable and ΔCBFΔSaO2 or ΔLactateΔSaO2 as the regressor (shown as partial regression plots in Figure 5). ΔCBFΔSaO2 and ΔLactateΔSaO2 were calculated as the change in the parameters between the normoxia and hypoxia measurement divided by the amount of desaturation (Equation 3).


(Eq. 3) ΔCBFΔSaO2=CBFHypoxia−CBFNormoxiaSaO2,Normoxia−SaO2,Hypoxia;ΔLactateΔSaO2=LactateHypoxia−LactateNormoxiaSaO2,Normoxia−SaO2,Hypoxia

Sex, age, and the proportion of gray matter in the MRS voxel were added as covariates.

## 3. Results

The total brain volume, brain parenchymal fraction, total gray matter volume and total white matter volume for each group are demonstrated in Figure 2 and summarized in Table 2. Patients with T1D and albuminuria had lower total brain volume, brain parenchymal fraction and gray matter volume than healthy controls and patients without albuminuria. The resting values of CBF, CMRO_2_ and lactate concentration are provided in Table 2. There were no significant differences in resting values among the three groups.

Figure 3 demonstrates the course of SaO_2_, end-tidal CO_2_ and heart rate during the inspiration of hypoxic air. The groups had similar courses for all parameters with an average desaturation to approximately 78% at the lowest. End-tidal CO_2_ decreased for all groups due to increased ventilation during the challenge. The heart rate increased slightly in all groups.

The effect of hypoxic challenge on cerebral physiology is shown in Figure 4. CBF and cerebral lactate increased significantly in all groups following the inhalation of hypoxic air and CMRO_2_ was unaffected in all groups. However, we observed differences in the strengths of the responses to the hypoxic challenge between patients with T1D and albuminuria and the healthy controls. Patients with T1D and albuminuria had a significantly reduced lactate increase ( β =0.008 mmol/l% (CI = [0.007; 0.015]), *p* = 0.032) compared to healthy controls and a borderline reduced response compared to patients without albuminuria ( β =0.006 mmol/l% (CI = [−0.001; 0.013]), *p* = 0.085) indicating a reduced glycolytic capability (Figure 4D). The patients with T1D and albuminuria had a lower CBF response ( β =0.20 (ml/100g/min)% (CI = [−0.13; 0.52]), *p* = 0.24) than healthy controls, however not significantly different (Figure 4D). We did not observe any differences between the patient group without albuminuria and the healthy control group (Figure 4D).

A small lactate response ( ΔLactateΔSaO2 ) to hypoxic air exposure correlated significantly with a reduced total brain volume ( β =3,812 ml/ΔLactateΔSaO2 (CI = [1,389; 6,235]), *p* = 0.003) and gray matter volumes ( β =1,405 ml/ΔLactateΔSaO2 (CI = [316; 2,495]), *p* = 0.013) across all subjects (Figure 5). If only the patients were included in the regression model, the correlation remained significant for total brain volume ( β =4,132 ml/ΔLactateΔSaO2 (CI = [685; 7,578]), *p* = 0.020) but not for gray matter volume ( β =1,252 ml/ΔLactateΔSaO2 (CI = [−238; 2,741]), *p* = 0.096). The CBF response to hypoxia ( ΔCBFΔSaO2 ) did not correlate with brain sizes (Figure 5).

## 4. Discussion

In the present study, we tested whether reduced CVR or hindered cerebral glycolytic activity could be observed in patients with T1D and whether this could be related to brain atrophy. We did this by measuring the CBF, CMRO_2_ and lactate response to a hypoxic challenge in human T1D patients using MRI and MRS techniques. By using a hypoxic challenge, we could quantify both cerebrovascular function and the capability for upregulating glycolysis.

The patients with T1D and albuminuria had significantly lower total brain volume, brain parenchymal fraction and gray matter volume than the control group and T1D patients without albuminuria. The patients with albuminuria also had significantly lower lactate response to the hypoxic challenge, demonstrating an abnormal cerebral physiological response to metabolic stress. We did not observe abnormal brain atrophy or cerebral physiological deficit in patients without albuminuria. Albuminuria is an indicator of vascular damages and a higher allostatic load from diabetes disease and the observation of atrophy and physiological deficits only in patients with albuminuria suggests that these deficits are linked to the severity of diabetes disease.

Reduced CVR to vasodilatory stimuli has previously been observed in T1D patients (Fulesdi et al., 1997). These studies used the inspiration of air with added CO_2_ compared to the inspiration of hypoxic air used in the present study. Episodes of hyperglycemia damage the vessel endothelium cells and hinder the nitric oxide mediated relaxation of the vascular smooth muscle cells, reducing the vasodilating capabilities and causing reduced CVR (Creager et al., 2003). Endothelial damage also increases the risk of atherosclerosis and cardiac disease and is part of the pathophysiology of typical diabetes complications such as nephropathy, neuropathy, and retinopathy (Rossing et al., 1995; Nin et al., 2011; Thomas et al., 2015; DiMeglio et al., 2018). Impaired CVR is also associated with neurodegenerative diseases and cognitive decline (Silvestrini et al., 2006). In our study, we did not observe inhibited CVR or impaired CMRO_2_ to the hypoxic challenge or correlation between reduced CVR and brain atrophy in the T1D patients. The missing correlation between impaired CVR and total brain volume and gray matter volume in combination with the maintained CMRO_2_ suggests that endothelium dysfunction is not a primary driver of brain atrophy, at least not at the disease stages observed in our patients. If we examined at a later and more severe disease stage, we might have observed more affected CVR and vascular deficits, which could contribute to the development of neurodegeneration similar to what is observed in vascular dementia.

In contrast to CVR, we observed that a reduced lactate response in patients with albuminuria correlated with smaller brains, suggesting glycolytic failure as a potential cause of brain atrophy. Importantly, we did not observe a reduced CMRO_2_, demonstrating that the lactate response to the hypoxic challenge is not a result of hindered oxygen metabolism but rather an upregulation of glycolytic activity. The normal healthy response to an inhalation of hypoxic air is a robust increase in the cerebral lactate concentration even though oxygen metabolism is maintained (Kety and Schmidt, 1948; Cohen et al., 1967; Harris et al., 2013; Vestergaard et al., 2016, 2022). The lactate response does not derive from an increase in systemic lactate, as previous studies using the same experimental setup as the present study found that the hypoxic challenge does not significantly increase systemic blood lactate (Vestergaard et al., 2016). Hence, the exact cause for the increased cerebral lactate production is not obvious; however, a likely mechanism is through the HIF-1 signaling pathways, which are the main regulators of oxygen metabolism in the cell (Ratcliffe et al., 1998). Reduced oxygen concentrations cause an accumulation of HIF-1 in the cell, which initiates numerous adaptive mechanisms, including mechanisms related to the upregulation of glycolysis, glycolytic enzymes and glucose consumption (Kim et al., 2006). The cause for a reduced stimulated glycolytic activity in patients with T1D could be due to GLUT downregulation in the brain. Preclinical studies have observed that hyperglycemia reduces GLUT in brain tissue (Wei-kai et al., 2007). Downregulation of GLUT hinders the influx of glucose into brain tissue. This could be an adaptive mechanism for protection during hyperglycemic episodes to avoid excess glucose entering the brain tissue, which can be neurotoxic and cause neurodegeneration. This adaptation, however, could be disadvantageous when the brain needs to increase glucose consumption during normoglycemia, for example, in response to hypoxic air exposure, where the response then is blunted.

Another cause for a reduced glycolytic response to hypoxia could be alterations in HIF-1 pathways, which are complexly involved in many complications to diabetes. Insulin increases HIF-1 through phosphoinositide 3-kinases and target of rapamycin pathways (Doronzo et al., 2006), and depletion of insulin therefore reduces HIF-1 expression. Furthermore, hyperglycemia destabilizes the HIF-1 molecule and represses the transactivation activity of HIF-1 and related pathways (Treins et al., 2002; Gunton, 2020). Abnormal oxygen metabolism is likely part of the pathophysiology in many diabetes-related comorbidities, for example, in diabetic nephropathy where the renal tissue becomes hypoxic (Franzén et al., 2016; You-Zhen et al., 2020; Laursen et al., 2021; Packer, 2021). An altered HIF-1 response to this hypoxia could be a part of the pathology, as increasing the expression of HIF-1 by prolyl hydroxylase domain protein inhibitors improves kidney function in a mouse model of diabetic nephropathy (Sugahara et al., 2020). HIF-1 is also impaired in a mouse model of diabetic wounds and increasing the expression of HIF-1 improves the wound healing process (Ruxandra Botusan et al., 2008). HIF-1 dysregulation will also promote atherosclerosis and cardiac disease, which are frequent complications in diabetes (Semenza, 2014). Glycolytic dysfunction has also been observed in the cardiac tissue of patients with diabetes, and the overexpression of HIF-1 in a mouse model of diabetes improved cardiac function and outcomes (Stanley et al., 1997; Xue et al., 2010). Pancreatic beta cell failure in type 2 diabetes could also be related to HIF-1 dysfunction. During hyperglycemia, beta cells become hypoxic, and an insufficient response from HIF-1 pathways likely plays a role in the development of beta cell failure (Cheng et al., 2010). Last, the HIF-1 gene Pro582Ser polymorphism, which increases HIF-1 activity, protects against diabetic nephropathy and retinopathy and seems to lower the risk of developing type 2 diabetes (Yamada et al., 2005; Gu et al., 2013).

A potential dysfunction of the HIF-1 pathways would also affect the brain. Tissue hypoxia with consequent disruption of energy homeostasis is likely a key component in AD and neurodegenerative diseases. Furthermore, an inability to maintain a correct balance between glycolysis and oxidative metabolism, as regulated by HIF-1, seems to be an important component in the development of AD (Vlassenko et al., 2010; Zhang et al., 2021). Correspondingly, an upregulation of the glycolytic enzymes mediated by HIF-1 has been shown to protect against AD pathology in both animal models and postmortem brain tissue from patients with Alzheimer’s disease (Newington et al., 2012; March-Diaz et al., 2021). HIF-1 also regulates GLUT in the brain, and reduced HIF-1 activity results in lower GLUT density (Chavez et al., 2000). Overall, these results indicate that HIF-1 dysfunction can reduce glycolytic activity in the brain and that such impairment can be associated with neurodegeneration. This corresponds to the reduced lactate response in patients with T1D and albuminuria and its correlation with smaller brains observed in our results.

### 4.1. Strength and limitations

The main strength of the study was that we measured CBF, CMRO_2_ and cerebral lactate concentration nearly concurrently and during rest and in response to a hypoxic challenge. This allowed us to examine both the cerebrovascular function and glycolytic capability.

The technique for measuring CBF has been validated in both human and animals against ^15^O-water positron emission tomography (PET) imaging (Vestergaard et al., 2017; Ssali et al., 2018; Puig et al., 2019). Measurement of global CMRO_2_ using the SBO MRI technique has been validated against measurement from blood samples acquired by catheter from the jugular vein during MRI scanning (Miao et al., 2019). The validation was performed both at normoxia and during hypoxic conditions.

A limitation of the study was the small sample size. The limited sample size could be a reason for why the smaller CBF response in patients with albuminuria was not significantly different from healthy controls. Nevertheless, we do not consider this to be a substantial shortcoming of the study, as it was apparent that the reduced CVR was not severe enough to affect CMRO_2_, which is the important parameter to describe a potential oxygen metabolism deficit. Including more subjects in the study would most likely not affect this conclusion as the CMRO_2_ was highly stabile with no trends towards an effect from hypoxia. Also, the sample size was sufficient to observe a significantly reduced lactate response in the patients with albuminuria.

A further limitation is that we only indirectly examine the glycolytic activation by measuring lactate response. Also, we do not directly measure HIF-1 or GLUT density in the brain, which could be the underlying cause for a reduced glycolytic activity. These parameters are not possible to measure *in vivo* in human brains, and an indirect approach by measuring brain lactate concentration was therefore a feasible alternative.

## 5. Conclusion

From the findings of the present study, we propose a new possible pathway causing harmful neurodegeneration in patients with T1D. We observed a limited capability to upregulate glycolytic activity, as evidenced by a smaller lactate response to hypoxic air exposure. This dysfunction correlated with smaller brains and reduced gray matter and was observed before severe cerebrovascular dysfunction. These deficits were only observed in patients with albuminuria suggesting that the deficits are linked to the severity of diabetes disease. We speculate that this reduced glycolytic capability may ensue from a reduced GLUT density or HIF-1 activity in the brain from insulin depletion or hyperglycemia. A reduced cerebral glycolytic activity could be a driver of neurodegeneration and be related to the increased risk of dementia development. Counteracting the reduction in cerebral glycolytic activity could be a possible point of intervention for preventing detrimental neurodegeneration in patients with T1D.

### Code availability

Software used to calculate blood flow from the PCM technique is available at https://github.com/MarkVestergaard/PCMCalculator/. Software used to calculate venous oxygen saturation from the SBO technique is available at https://github.com/MarkVestergaard/SBOCalculator/.

## Data availability statement

The parameters derived from the MRI-images are available from the corresponding author upon reasonably request. The MRI images are not publicly available due to privacy restrictions.

## Ethics statement

The studies involving humans were approved by The Capital Region of Denmark Committee on Health Research Ethics. The studies were conducted in accordance with the local legislation and institutional requirements. The participants provided their written informed consent to participate in this study.

## Author contributions

MV and HL initiated and formulated the study. JL and NH recruited the participants. JL, NH, TH, and PR performed the clinical evaluation and characterization of the participants. MV, JL, and NH acquired the data. MV performed the data processing, statistical analysis, and wrote the article. All authors provided interpretations of the results and have approved the article.
